# Editorial

**Published:** 1839-08-24

**Authors:** 


### THE MEDICAL EXAMINER.

### PHILADELPHIA, AUGUST 24, 1839.

We extract a part of a letter from our friend, Dr. Paul F. Eve, to the Editor of the Southern Medical and Surgical Journal, relative to the statistics of amputations in the Pennsylvania Hospital. We regret that we differ in many respects from the writer.

The statistics of amputations published by Dr. Norris are, we believe, the only ones published in this country; they embrace a period of several years, and were performed by men of high surgical attainments in our admirably conducted hospital. Now, what do they prove? Simply that the mortality after amputation, under these circumstances, is much greater than is generally supposed; and, therefore, the operation is dangerous in the hospitals of America, as well as in those of Paris, although to a less degree in the former than in the latter. Before these statistics were published, the impression in the minds of most surgeons at Philadelphia was, that amputations were almost always successful. This was the conviction of the surgeons of the Pennsylvania Hospital as well as of others, and is probably the universal belief in the minds of men who rely on recollection only. A few years since, when we were connected with the hospital, a friend of ours at the Hôtel Dieu of Paris, wrote to inquire what was the proportionate mortality at the two hospitals. By looking over the cases, we found that with us it was at that time extremely large, and that the patients were either carried off by purulent absorption and its attending fever, or died of the immediate consequences of the operation, or of the disease which had required it. The mortality at that time was much above the average; nevertheless, it is never inconsiderable in patients placed under similar circumstances.

The only circumstances unfavourable to the recovery of the patients at the Pennsylvania Hospital, are—1st, the peculiar influence of the atmosphere of the institution, which occasionally gives rise to erysipelas and to purulent absorption, which is in some way connected with it; and, 2d, the late period at which the surgeons perform some of the amputations.

As to the first cause, it will, in all probability, never be entirely removed, but may be obviated, in a considerable degree, by avoiding operations, unless of indispensable necessity, where erysipelas prevails. The second cause is altogether a matter of opinion; there does exist amongst the surgeons of Philadelphia, a strong repugnance to the performance of amputations. This, in itself, is most praiseworthy, and undoubtedly is the means of saving not a few limbs, which would otherwise be lost; it may be, that it is occasionally carried too far. Nevertheless, the motives which dictate it are in accordance with humanity and sound surgical science.

The comparison with the French hospital practice and that of the American surgeons, is often made,—always greatly to the disadvantage of the former. We are quite aware that there are well grounded objections to some points of practice connected with French surgery; still, the great causes of the mortality after simple surgical operations, such as amputations, are certainly the great size of the wards in which the patients are placed, or at least the imperfect ventilation which is there practicable, and the miserable constitutions of many of the subjects. It was a matter of common remark to us, that the mortality was much less at the hospitals of the provincial towns than those of Paris, and less considerable at the hospitals situated in the suburbs of Paris, than those of the central parts of the town. This fact, in a great degree, explains the causes of the mortality, and proves that it is unjust to compare the success in private practice with that met with in the patients of our hospitals.

The statistics of Dr. Norris are of great value, because they are drawn up in accordance with strict observation, and bring out the unsuccessful cases into prominent relief, instead of keeping them carefully out of sight. Hence, full reliance may be placed upon his conclusions.

The very favourable reports of surgical practice which are occasionally published in this country, have always been drawn up, as far as we know, with the design of giving the true results; but we fear that the sensitive dread of confessing the failure of operations, has sometimes led surgeons to deceive themselves as to the results,—that is, they forget the unsuccessful cases, and remember only those which terminated well.

At least, this is the conclusion we formed from the reports of Dr. Norris, which, if we are not mistaken, were different from the impression which the author had previously received.

"M. Velpeau, in preparing the second edition of his Medicine Operatoire, wrote to Dr. Mott, requesting him to give some idea of the success of American surgeons. This Dr. Mott soon furnished; but M. Velpeau, I learn from his chief interne, M. Perischaud, does not give credit to it. He says this is contradicted by the statistics of Dr. Norris, one of the surgeons of the Pennsylvania Hospital. I recollect being impressed with the great error which Dr. Norris' statement was calculated to produce, by those who take it as the basis of success of amputations in the United States. It no more conveys a correct history of American surgery on this, than it does on any other subject. No surgeon of our country will consent to its being a correct foundation of statistics in surgical practice. All it can pretend to, and all that Dr. Norris undoubtedly intended by it, was the practice of the Pennsylvania Hospital, and nothing more. I respect the surgeons of this charitable institution, but I am sure they even willacknowledge that they erred, and that greatly, though on the side of mercy, in delaying amputations during the period referred to by Dr. Norris. Who, in reading these statistics, will admit them as correct, as applied to the United States? And these being the only ones yet published in our country, it is not astonishing that a man of M. Velpeau's industry and penetration, should have noticed the contradiction to it in Dr. Mott's letter to him."

### CHARACTER OF THE DISEASES OF THE PRESENT SEASON AT PHILADELPHIA.

The present season has offered no prevailing disease, other than those which are incident to the summer, such as cholera infantum, dysentery, and intermittent and remittent fever. The cases of remittent and intermittent have thus far been decidedly more numerous than they have been for several years past, and all that have offered themselves in our practice have been characterized by an extraordinary degree of prostration and feebleness of both nervous and circulatory system. The pulse is uniformly slow throughout a great part of the affection, but especially at the period of remission and of intermission, when it sinks to fifty or sixty in the minute. In no one case that we have yet seen, has the pulse offered the character of an inflammatory disorder, or one characterized by a strong, laboured action of the vascular system.

We have thus far treated about twenty cases without loss, nor have they generally been protracted. Some of them terminated in eight days, others at the end of a fortnight.

The mode of treatment we have found necessary is in accordance with the peculiar constitution of the period. In no one case was bleeding necessary; on the contrary, it always seemed inappropriate; purging was directed very moderately, and at the commencement. The purgative was generally calomel, combined with rhubarb. The purgative was repeated in a very small proportion of cases, and, after the first dose, consisted simply of a light laxative.

During the course of the disease, the remedies consisted of the mild diaphoretics, especially the spirit of mindererus, and preparations of quinine, as early as possible. The quinine did not agree with some of the patients in whom the disease was less distinctly remittent; but by suspending its use for a day or two, and again recurring to the diaphoretics, we were always able to resume it with good effect. We have not treated any case of a remittent form without quinine, but the milder cases would doubtless have passed off without any specific treatment. When the liver and spleen were much enlarged, a grain of blue mass was combined with two grains of quinine, and given every one or two hours.

The patients, after their recovery, remained for a long time extremely feeble, and, as usual, the spleen and liver were somewhat enlarged. These symptoms were combated by quinine and some preparation of iron, such as the carbonate and phosphate, and occasionally by infusions of serpentaria and cinchona. When gastritis occurred as a complication, it was removed by the usual remedies, such as cups to the epigastrium, &c.

It is extremely probable that the constitutional tendency of the remittent diseases of this summer extends much further than Philadelphia, and much injury may result from adopting too active, or, rather, too debilitating a treatment. With us the fevers in a few cases were attended with so much prostration as to require wine-whey, or some stimulant of the kind, in addition to the preparations of cinchona. We should be glad to learn from our correspondents the results of their experience on this subject.

